# Deep learning-based approach for 3D bone segmentation and prediction of missing tooth region for dental implant planning

**DOI:** 10.1038/s41598-024-64609-0

**Published:** 2024-06-16

**Authors:** Mohammed Al-Asali, Ahmed Yaseen Alqutaibi, Mohammed Al-Sarem, Faisal Saeed

**Affiliations:** 1 College of Computer Science and Engineering, Taibah University, 42353 Medina, Saudi Arabia; 2 Substitutive Dental Sciences Department (Prosthodontics), College of Dentistry, Taibah University, 41311 Al Madinah, Saudi Arabia; 3 Department of Prosthodontics, College of Dentistry, Ibb University, 70270 Ibb, Yemen; 4 Department of Computer Science, Sheba Region University, Marib, Yemen; 5 College of Computing and Digital Technology, Birmingham City University, Birmingham, B4 7XG UK

**Keywords:** Computer science, Oral anatomy

## Abstract

Recent studies have shown that dental implants have high long-term survival rates, indicating their effectiveness compared to other treatments. However, there is still a concern regarding treatment failure. Deep learning methods, specifically U-Net models, have been effectively applied to analyze medical and dental images. This study aims to utilize U-Net models to segment bone in regions where teeth are missing in cone-beam computerized tomography (CBCT) scans and predict the positions of implants. The proposed models were applied to a CBCT dataset of Taibah University Dental Hospital (TUDH) patients between 2018 and 2023. They were evaluated using different performance metrics and validated by a domain expert. The experimental results demonstrated outstanding performance in terms of dice, precision, and recall for bone segmentation (0.93, 0.94, and 0.93, respectively) with a low volume error (0.01). The proposed models offer promising automated dental implant planning for dental implantologists.

## Introduction

Dental implants are widely recognized as a prevalent and primary treatment modality for individuals suffering from tooth loss. Ongoing research endeavors are diligently focused on advancing the field of implantology, with the ultimate goal of enhancing treatment outcomes and establishing long-lasting and visually pleasing restorative solutions^1–4^.

Dental implant placement is a meticulous process that requires careful planning to minimize surgical risks and achieve the best esthetic and functional results^2^. This planning includes several sequential stages, such as identifying the edentulous area (also known as the missing tooth region that refers to the space in the dental arch where one or more teeth have been lost), locating and evaluating vital anatomic structures, measuring bone dimensions, and virtually placing the dental implant using specialized planning software programs^3,4^.

Numerous studies have utilized cone-beam computerized tomography (CBCT) images to aid dental implant placement and planning^5–11^. For example, Peck and Conte^11^ demonstrated that treatment planning based on CBCT and 3D multiplane treatment procedures can improve decision-making for complex implant placements. Various technologies have been employed to enhance the accuracy of dental implant procedures, including surgical guides, 3D printing, augmented reality, virtual reality, navigation systems, and artificial intelligence (AI). With the advent of the artificial intelligence revolution, intelligent methods have emerged as powerful tools for diagnostic analysis of CBCT scans. These methods offer insights for treatment planning by suggesting optimal implant positions and sizes based on patient history and anatomical data. While the applications of AI for dental implant procedures are still expanding, current areas of focus include implant type recognition, prediction of implant success, optimization of implant design, and prediction of implant survival^12,13^.

Machine learning systems, which utilize training data sets to recognize patterns, have been widely applied for implant planning. Deep learning is an increasingly popular variant of machine learning based on artificial neural networks that have been widely used in various applications^14^. Several studies applied deep learning models, such as convolutional neural networks (CNN), for implant-type recognition using radiographical data like periapical and panoramic images^13,15–18^.

One of the primary hurdles in implant planning is accurately determining the location of missing teeth. Deep learning methods offer a means to enhance this prediction’s accuracy by precisely segmenting the missing tooth bone regions in CBCT scans. Manual segmentation methods for implant placement are time-consuming and inefficient, making integrating deep learning models crucial for improving the accuracy and efficiency of this critical task. This paper presents a deep learning-based approach to enhance the efficiency and precision of dental implantology. Two U-net-based deep learning models were developed: one for segmenting the region of interest (ROI) of missing tooth bone on CBCT scans and another model for predicting the implant position. The main contributions of this manuscript can be summarized as follows:

- 1. Compiling an authentic dataset of images derived from confidential patients’ CT scans that depict various missing teeth across diverse age groups. The CBCT images were collected from patients aged 16 to 72, with ethical clearance obtained from Taibah University’s College of Dentistry, Madinah, Saudi Arabia (approval # 14032021, granted on May 8, 2022).
- 2. Implementing the first U-net-based deep learning model for segmenting the region of interest (ROI) of missing tooth bone in CBCT scans.
- 3. Implementing the second U-net-based deep learning model for implant position prediction. A domain expert verified the reliability of the dataset for future dental planning and implantation manually.
- 4. The utilization of the U-net model outputs (predicted 3D volumes) to generate stereolithography (STL) files that can be printed in 3D format.
- 5. Testing the proposed models on several new cases, with the results confirmed through manual verification by a domain expert, to ensure the reliability and efficiency of the proposed models.

## Results

**Figure 1:**
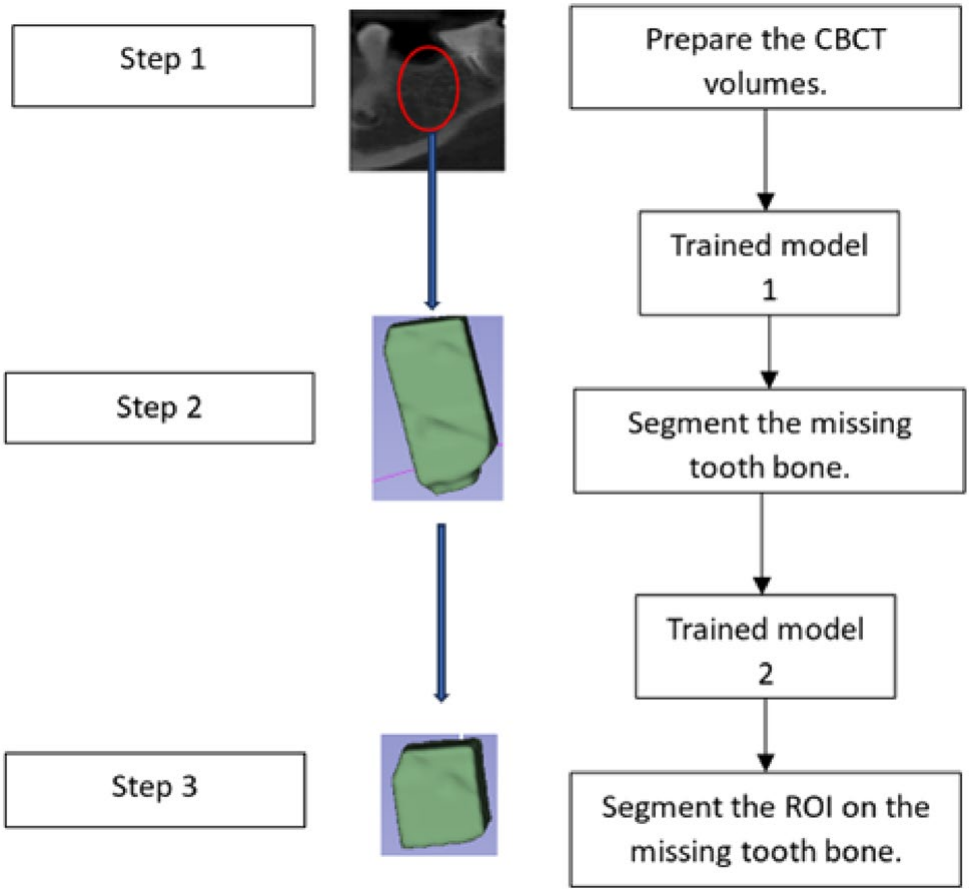
Experimental setup for the proposed U-NET models.

This study aims to propose U-Net deep learning models for segmenting missing tooth bone regions in CBCT scans and predicting the implant position. The U-Net models undergo training and testing in three stages. After preparing the CBCT volumes, the proposed procedure starts with segmenting the missing tooth bone from the entire volume using the first U-Net model, as shown in Fig. 4 (step 2). Then, the second U-Net model is used to segment the region of interest (ROI) on the missing tooth bone, enabling the detection of the suitable implant region (Fig. 1, step 3).

### U-Net model 1: segmenting the missing tooth bone from the entire volume

We utilized the U-net model to improve the process of segmenting the specific region of interest for tooth implant planning without compromising prediction accuracy. The configuration and hyperparameter tuning of the model are explained in the Sect “Methods”. We evaluated the performance of the proposed model using various metrics, including dice, Jaccard, precision, recall, false positive rate (FPR), false negative rate (FNR), “VS” (volume of similarity), “HD” (Hausdorff distance), “MSD” (mean square distance), “StdSD” (standard deviation of the surface distance), and “HD95” (the 95th percentile of the surface distance). More details about these metrics can be found in the supplementary material. The definition and description of these metrics are summarized in Table 1.

**Table 1:** Metrics for measuring effectiveness of AI models.

Metrics	Description	Calculation formula
Dice similarity coefficient	Measures the overlap between the ground truth and predicted segmentations, where a value of 1 indicates a perfect overlap and a value of 0 indicates no overlap	Dice = (2 * |A ∩ B|) / (|A| +|B|) Where A and B are the binary arrays or sets being compared. |A| represents the cardinality (number of elements) of set A, and |A ∩ B| represents the number of elements common to both sets A and B
Jaccard coefficient	Measures the similarity between the ground truth and predicted segmentations, where a value of 1 indicates identical segmentations and a value of 0 indicates completely different segmentations	Jaccard =|A ∩ B| / |A ∪ B|
Precision	Measures the proportion of true positives among all positive predictions, where a value of 1 indicates perfect precision and a value of 0 indicates no precision	Precision = TP / (TP + FP) TP refers to true positives, which are the correctly predicted positive instances. FP refers to false positives, which are instances predicted as positive but are actually negative
Recall	Measures the proportion of true positives among all ground truth positives, where a value of 1 indicates perfect recall and a value of 0 indicates no recall	Recall = TP / (TP + FN) FN refers to false negatives, which are instances that are actually positive but are predicted as negative
False positive rate (FPR)	Measures the proportion of false positives among all negative predictions, where a value of 0 indicates perfect specificity and a value of 1 indicates no specificity	FPR = FP / (FP + TN)
False negative rate (FNR)	Measures the proportion of false negatives among all ground truth positives, where a value of 0 indicates perfect sensitivity and a value of 1 indicates no sensitivity	FNR = FN / (TP + FN)
Volume of similarity (VS)	Measures the similarity between the volumes of the ground truth and predicted segmentations, where a value of 0 indicates identical volumes and a value of 1 indicates completely different volumes	VS =|A ∩ B|
Hausdorff distance (HD)	Measures the maximum distance between any point in one segmentation and its nearest point in the other segmentation	HD(A, B) = max(h(A, B), h(B, A)) h(A, B) represents the directed Hausdorff distance from A to B, and h(B, A) represents the directed Hausdorff distance from B to A
Mean square distance (MSD)	Measures the mean distance between the points in one segmentation and their nearest points in the other segmentation	MSD = (1 / N) * ∑(d^2) N is the total number of points or voxels, and d is the Euclidean distance between corresponding points or voxels in the two datasets
Standard deviation of the surface distance (StdSD)	Measures the standard deviation of the surface distance between the ground truth and predicted segmentations	StdSD = sqrt((1 / N) * ∑((d—mean(d))^2)) N is the total number of points or voxels, d is the surface distance between corresponding points or voxels in the two datasets, and mean(d) is the mean surface distance
The 95th percentile of the surface distance “HD95”	HD95 represents the 95th percentile of the surface distance distribution. It indicates the surface distance below which 95% of the points or voxels fall	

The results demonstrate that the model achieved moderate values for dice, Jaccard, precision, and recall, as shown in Table 2. Additionally, the volume error rate was relatively low (14.32%). These findings suggest that the predicted segmentation shows moderate overlap and similarity with the ground truth segmentation.

**Table 2:** Performance of first trained U-net model.

Dice	Jaccard	Precision	Recall	FPR	FNR	VS	HD	MSD	StdSD	HD95	Volume error rate
0.81	0.68	0.87	0.75	0.00	0.25	0.15	2.96	0.38	0.41	1.23	14.32%

**Figure 2:**
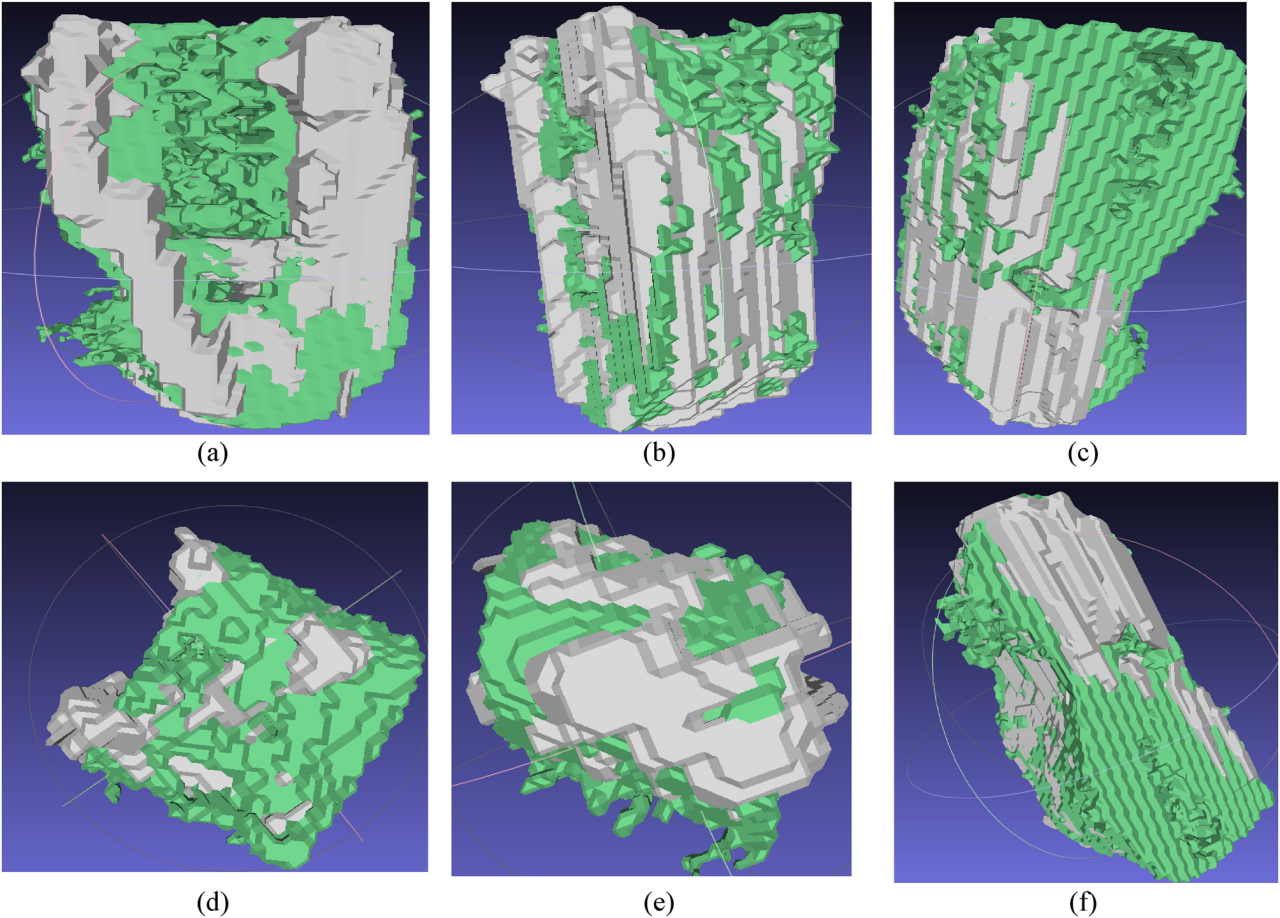
Side view of the volume obtained by the first trained model: (**a**–**c**) front side view of the volume of tooth bone, (**d**) upper view of the volumes, (**e**) bottom view of the volumes, (**f**) side view from the bottom of the volumes.

To visualize the results of this model, we used the MeshLab software to overlay STL files created for both the mask (ground truth) and predicted volumes. Figure 2 shows three side views of a single volume. The silver color represents the predicted volume, while the green color represents the ground truth mask.

### U-Net model 2: segmenting ROI for implant planning in the missing tooth bone

This model’s configurations and evaluation metrics are similar to those of the first model. However, a domain expert was invited to annotate the edentulous area (the specific region for implant placement) as the ground truth (GT). Specifically, the expert used standard measures for dental implants, with a diameter of 4.1 mm and a length of 10 mm. The performance metrics of this U-net model are shown in Table 3. These results demonstrate high performance in terms of dice, Jaccard, precision, and recall values (0.93, 0.88, 0.94, and 0.93, respectively), with a low volume error rate (1%) for segmenting the ROI for implant planning in the missing tooth bone. These metrics suggest that the predicted segmentation has a high degree of overlap and similarity with the ground truth segmentation, with a relatively low false-positive rate, high precision, and recall.

**Table 3:** Performance of second trained U-net model.

Dice	Jaccard	Precision	Recall	FPR	FNR	VS	HD	MSD	StdSD	HD95	Volume error rate
0.93	0.88	0.94	0.93	0.00	0.07	0.01	2.41	0.18	0.27	0.66	1%

**Figure 3:**
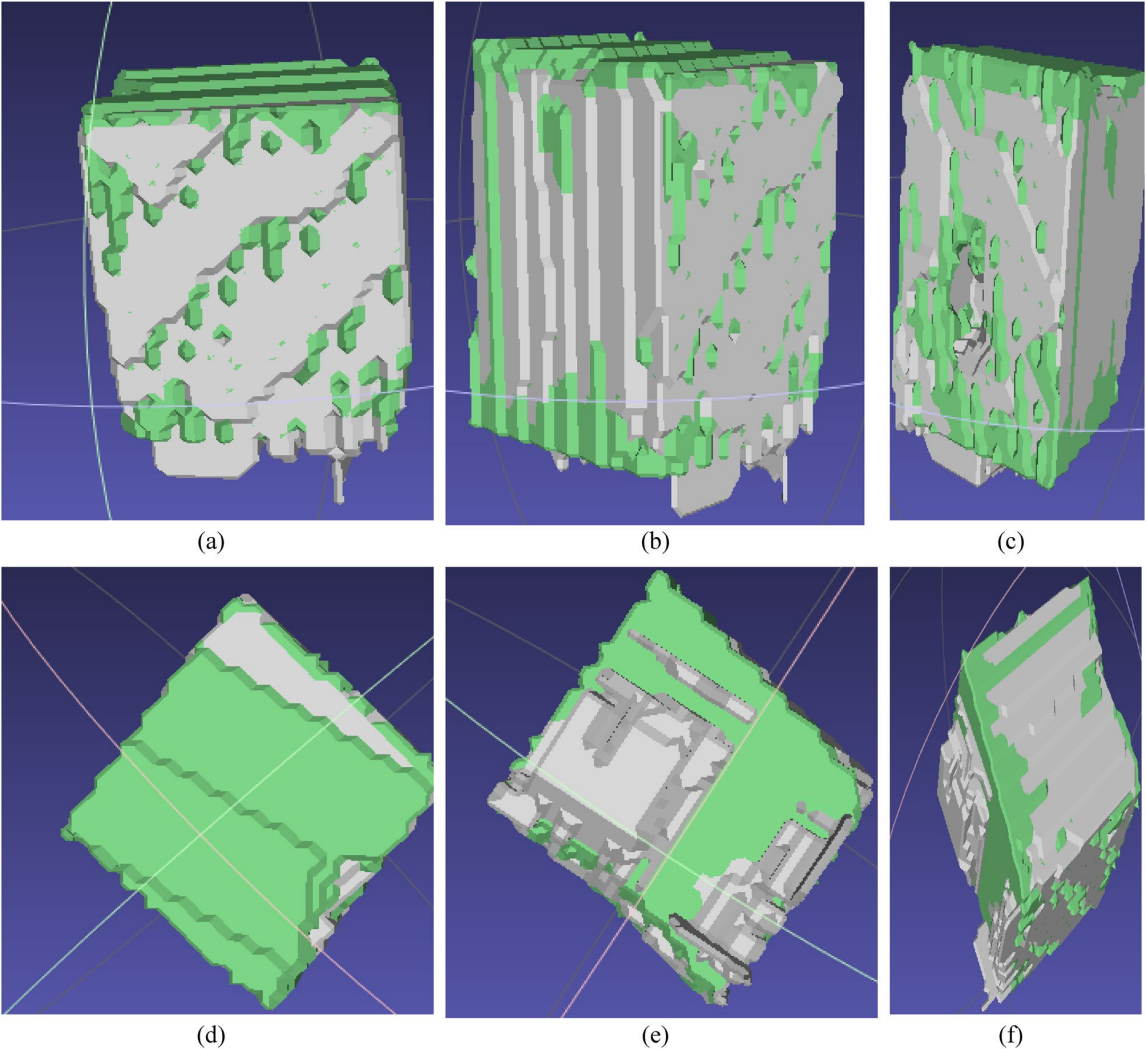
Side view of the volume obtained by the second trained model: (**a**–**c**) front side view of the volume of tooth bone, (**d**) upper view of the volumes, (**e**) bottom view of the volumes, (**f**) side view from the bottom of the volumes.

**Figure 4:**
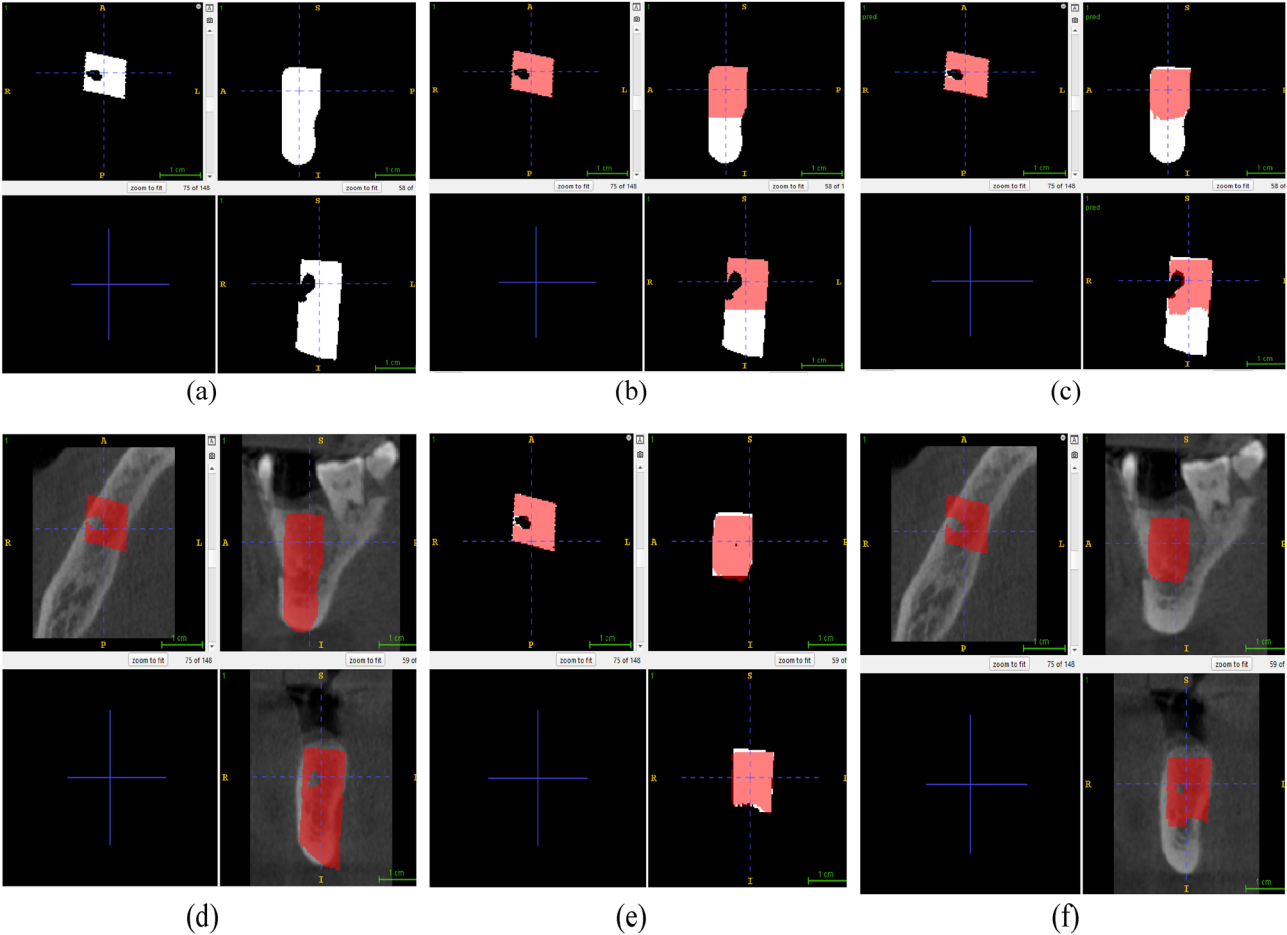
The mask augment to the volume using ITK-SNAP Software: (**a**) the ground truth mask of the missing tooth bone, (**b**) the ground truth mask of the implant augmented with the ground truth mask of the missing bone, (**c**) the predicted mask of the implant augmented with the predicted mask of the missing bone, (**d**) predicted mask of the implant augmented with the entire volume, (**e**) the predicted mask of the implant augmented to ground truth mask of the implant, and (**f**) the predicted implant mask augmented with the entire volume.

The output from the first model was used as an input for this model. Figure 3 shows the output of the second model from three different volume perspectives. The silver area represents the predicted mask, while the green area represents the ground truth mask. Figure 4 summarizes the output from both models. The CBCT volume was enhanced by overlaying the corresponding predicted masks, creating a visually improved representation.

To evaluate the performance of the proposed AI model, the domain expert was asked to annotate the used test dataset for the second U-net model, which involved drawing a bounding box around each area where a tooth was missing in the images. The annotations were made to compare with the ground truth data and determine how good the model was with respect to the expert’s opinion.

The predicted masks generated by this model highlight specific regions or structures of interest within the CBCT volume. This combined visualization offers valuable insights into the spatial distribution and localization of these structures, aiding in the analysis and interpretation of the CBCT data. It allows for a comprehensive understanding of volumetric information and facilitates more precise examination and diagnosis in various medical and imaging applications.

## Discussion

Artificial Intelligence has recently been used in dentistry for disease detection, classification, and organ and lesion segmentation^13,19–22^. Most published applications involve 2D radiographs, such as panoramic and periapical images, while limited studies used 3D radiographs like CBCT. Therefore, further advancements of AI in this area would greatly benefit dentists, allowing them to use computer-aided systems for 3D imaging procedures that require knowledge and expertise. Treatment planning is a critical stage in the medical and dental fields, where an accurate diagnosis is the foundation for personalized treatment strategies. Alongside the physician's education and experience, various other factors contribute to the treatment planning process^23^.

Volumetric 3D data in dentistry presents unique challenges for tooth segmentation and classification tasks. Dental segmentation is quite challenging due to several factors. Firstly, dental data often contains intricate structures like teeth, roots, and small bone structures, which makes it difficult to perform accurate segmentation due to their small size and complex shapes. Another challenge is the limited contrast and presence of noises in dental images, such as CBCT scans. This can make distinguishing between different tissues or structures difficult, resulting in lower segmentation accuracy. Dental restorations like fillings, crowns, and implants can introduce further segmentation challenges. These artificial structures can have different materials and shapes that may interfere with the segmentation process. Moreover, dental anatomy and conditions can vary significantly between individuals, adding complexity to the segmentation task as it requires handling diverse anatomical variations.

Dental implant planning heavily relies on radiographic imaging. 3D imaging equipment should be used to examine the surgical site before surgery, and a series of measurements should be taken under anatomical variation-permissive settings to assist in providing detailed planning^10,24^. The study assesses the vital anatomical differences that influence implant planning. Real-life cases were examined to test the effectiveness of the proposed U-Net model. Numerical analysis demonstrates that the proposed deep learning models can reduce volumetric errors to below 1%. The bone geometry of the final implant was also satisfactory, as depicted in Fig. 3, which presents the model output in 3D volume views. The results of the proposed models align with the manual measurements, suggesting the potential usability of the technology in implant planning.

Moreover, the proposed models are computationally efficient. The training, consisting of 200 epochs, required 58 min, and once trained, the prediction task only takes 10 s, making it more efficient compared to other approaches. Details of the computational settings and the training history of the proposed deep learning model are provided in the supplementary material.

As this study aimed to segment missing tooth bone using a conventional 2D UNET model, Moufti et al.^25^ reported some findings on using a 3D UNET from the MONAI framework. When compared to the results reported in Moufti et al.^25^, the dice values obtained in this work consistently outperformed those obtained in Moufti et al.^25^ The training dice consistently exceeded 90%, and the testing dice and total (average) dice were both higher than 90%. This suggests that the proposed model has better segmentation performance. In addition, a comprehensive comparison should consider the specific context and dataset. For instance, Bayrakdar et al.^26^ proposed 3D CNN model trained on annotated CBCT images to extract features and generate accurate implant plans automatically. The accuracy rates for detecting canals and sinuses/fossae were 72.2% and 66.4%, respectively. Park et al.^27^ focused on developing a deep learning-based system for detecting missing tooth regions in panoramic radiographic images. The authors train a deep learning model using a substantial annotated image dataset. This enables the model to learn patterns and features associated with missing teeth with 92.14% mean Average Precision (mAP) for tooth instance segmentation and 59.09% mAP for missing tooth regions detection. The application of transfer learning and artificial intelligence for guiding implant placement in the posterior mandible was also investigated by Lui et al.^28^ and Al-Sarem et al.^10^. Lui et al.^28^ conducted a vitro study and demonstrated the potential of pre-trained deep learning models for accurately predicting optimal implant positions and aiding dental professionals in achieving precise and effective implant placements. Al-Sarem et al.^10^ developed a deep learning-based model for detecting missing teeth positions in CBCT images, achieving high precision using pre-trained CNN models like DenseNet169. The model shows potential as a time-saving tool for automated dental implant planning.

Additionally, Alsomali et al.^29^ developed a deep-learning model that could automatically identify the precise location of gutta-percha (GP) markers on cone beam computed tomography (CBCT) images. These markers are utilized to designate potential dental implant sites. The researchers used 34 CBCT datasets, which consisted of images from patients wearing a radiographic stent during the imaging process for implant planning. These datasets were used to train, validate, and test the AI model. During the training process, the GP markers were manually marked on the axial images, which were then employed to train the deep learning model. The effectiveness of the AI model was evaluated using four CBCT datasets, and it achieved an 83% true positive rate in accurately identifying GP markers, with a false positive rate of 2.8%. Notably, 28% of the areas identified by the AI model as GP markers were incorrect, and 17% of the actual GP markers were not detected by the model. These findings highlight the limitations of relying solely on axial images to train an AI model and achieve accurate performance.

Similarly, Bodhe et al.^30^ explored the development of an AI-based system designed to assist medical professionals. This system determines implant types and positions, locates the mandibular canal, and identifies the total number of missing teeth. These capabilities aim to enhance the precision of dental implant procedures and maxillofacial surgeries, highlighting AI's potential to improve patient care by supporting decision-making in complex medical tasks.

While these previous studies provide valuable insights, it's crucial to mention that many of them focus on a broader range of oral and maxillofacial structures for different purposes, such as diagnosis and generalized implant planning. This work, however, specifically focuses on segmenting proposed implant site areas. This approach addresses unique challenges associated with this application, which may not be the main focus of the other previous studies. Therefore, our work provided precise and practical solutions that enhance the accuracy and reliability of implant site planning in clinical settings.

The importance of this research lies in its groundbreaking utilization of deep learning to enhance the precision and effectiveness of dental implant planning. The study highlights the potential of U-Net models as a useful tool in dental surgeries, specifically in precisely segmenting the area of interest for absent tooth structures in CBCT scans. Looking ahead, this research presents new opportunities for implementing AI in dental practices. It establishes the foundations for future developments that have the potential to enable real-time guidance during surgical procedures with AI assistance, as well as the integration of technologies such as augmented reality to enhance implant planning. The ongoing improvement of these AI models is anticipated to enhance patient outcomes in dental implantology significantly.

Several limitations need to be addressed in this study. Firstly, the deep learning model used for segmenting missing tooth bone has achieved only moderate accuracy. While the model assists in the process, it should aim for more optimal performance in predicting implant outcomes accurately. This implies that further improvements or refinements are necessary to enhance the model's accuracy. Secondly, the reliability and accuracy of the available software, such as the 3D Slicer, used for segmenting missing tooth bone may vary. While these software tools can offer some level of segmentation, there is a potential for introducing errors or inconsistencies in the final results when using this software. Another limitation is associated with segmenting the region of interest. Currently, the study relies on manually cropping volumes to isolate the region of interest for missing tooth segmentation. This process is time-consuming. Developing a machine learning model specifically designed to efficiently and accurately crop the volumes would be advantageous, leaving only the region of interest for missing tooth segmentation. Such a model would significantly improve the accuracy and consistency of the segmentation process.

## Methods

### Dataset acquisition

This study examines CBCT imaging of patients at Taibah University Dental Hospital (TUDH) between 2018 and 2023. CBCT images were obtained for patients aged 16–72. Low-quality images that could impact the accuracy of the deep learning models were excluded from the dataset. Images of individuals with a history of injury, illness, surgery, congenital conditions, fractures, or any other foreign bodies that may cause artifacts in the images were also removed. Additionally, fuzzy images with unclear bone borders were disqualified. Only 150 out of 890 reviewed scans met the requirements. The computer used was password-protected, each participant received a unique identifier, and the data were stored in a password-protected Excel spreadsheet. Technological factors like picture quality and CBCT reconstruction time can affect the visibility of anatomical details. To minimize variations in these factors, all CBCT scans in this study were performed at TUDH using the same machine (KaVo 3D eXam; KaVo) with the specified device settings (i.e., 120 kVp and 5 mA, using a field of view of 1613 cm, voxel size of 0.25 mm, and acquisition time of 26.9 s). The resolution of the CBCT images was standardized to 79 × 112 × 135 pixels, with a 0.3 mm gap between each image. CT data represents various bone and tissue types, and the imaging conditions differ among patients. A Hounsfield unit-based intensity threshold of [− 1000, 2000] was used to preserve the skeletal structure. Selecting the appropriate intensity threshold is crucial for successful segmentation. 48,900 images were obtained after each remaining volume was cut along the x, y, and z axes.

### Dataset organization

The collected images were divided into training and validation folders, with 20% allocated for validation and 80% for training. All images were resized to 24 × 24 and converted to grayscale. For testing, unseen volumes were sliced and stored in the testing directory. It's important to note that the test volumes include mask volumes for evaluating the model’s performance. Data augmentation was performed to generate a total of 61,088. Deep learning commonly employs this technique to expand and diversify a training dataset. It involves applying transformations or modifications to existing data samples, such as translations, scaling, flips, centering the feature distributions around zero (featurewise_center), normalization scales so that the images have a standard deviation of one (featurewise_std_normalization*)*, and specifying the range of angles within which an image can be randomly rotated (rotation_range). The primary objective of data augmentation is to enhance the generalization and robustness of deep learning models. Exposing the model to augmented versions of the original data aids in developing resistance to variations and noise that may arise in real-world scenarios and prevents possible overfitting. Featurewise_center was set to True to subtract the mean value from each sample, centering the data around zero and mitigating issues related to input feature scales. Featurewise_std_normalization was set to True to normalize the input data by dividing each sample by its standard deviation. This step scales the data to have zero mean and unit variance, aiding in convergence and performance improvement. The rotation_range was set to 90°, allowing for random rotations of the input images during data augmentation. Exposing the model to different orientations of the input data within the specified range makes it more robust to variations.

### Data labeling

**Figure 5:**
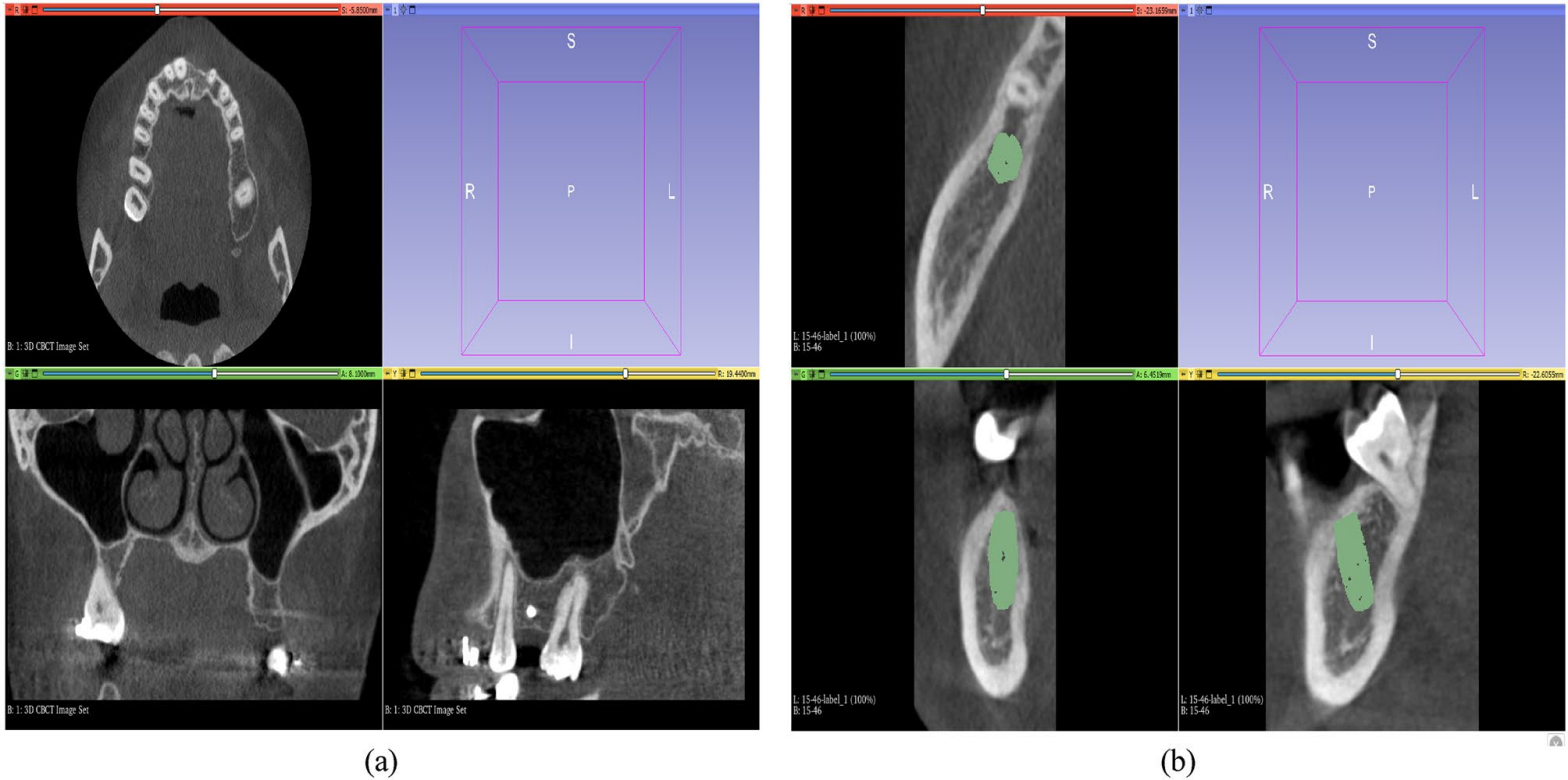
(**a**) 3D Slicer software windows after loading DICOM files of the patient’s jaw. (**b**)Implant planning axial, coronal, and sagittal sections.

To acquire the ground truth labels, the CBCT images of all patients were manually annotated on the 3D Slicer (4.7.0) software by one domain expert with more than 12 years of experience in dental implant planning. 3D Slicer is a versatile image-processing software widely used in the medical image-processing industry. The software employs automatic image segmentation and fusion techniques to generate 3D visual models from DICOM data. It also offers a transformation function. Head positions were standardized anteroposteriorly and sagittally using a standard method. Each jaw was separated into the anterior and posterior tooth regions and labeled as mandible or maxilla. Any missing teeth, canals, sinuses, or fossae in the alveolar bones were documented. Manual bone height and thickness assessments were performed in areas where teeth were missing. These assessments followed the same procedures as implant planning, considering anatomical constraints. Figure 5a displays the 3D Slicer software windows after loading the patient’s jaw DICOM files, while Fig. 5b showcases the implant planning axial, coronal, and sagittal sections.

To accurately simulate the placement of dental implants in edentulous regions using patient-specific anatomical data, we used a multi-step process with 3D Slicer software. The methodology included the following stages:

1. DICOM file preparation: initially, we imported DICOM files representing CBCT scans of patients into 3D Slicer. This step ensured that the patient-specific anatomical data were loaded accurately for further manipulation.
2. Segmentation: using the segment editor tool within 3D Slicer, we created a new segment to delineate the area of interest, specifically the edentulous region required for the implant. The segmentation process involved adjusting the threshold levels to match the bone density accurately, ensuring that the segment precisely represented the target anatomical structure.
3. Volume cropping: the cropped area representing the site for potential implant placement was refined using the crop volume module. This involved selecting and isolating the region of interest from the larger scan, focusing specifically on the area relevant to the implant placement.
4. Implant STL file integration: a pre-specified STL (stereolithography) file representing the dental implant configuration was then introduced into the working environment. This was done using the transform tool, allowing for precise placement and alignment of the implant model within the segmented and cropped bone region.
5. Refinement according to implant dimensions: the cropped bone segment was further refined to accommodate the dimensions of the implant model. This important step ensured that the virtual implantation was anatomically realistic and tailored to the specific dimensions of the patient’s site.
6. Finalization and export: After the implant was precisely positioned and its dimensions were adjusted within the bone segment, the implant model was removed. The final segment, representing the modified bone structure designed to receive the implant, was saved as a NIfTI file.

#### Quality assurance

A certified oral and maxillofacial implantologist with over 12 years of experience labeled and assessed all images. We randomly uploaded all data to a deep convolutional neural network to streamline the dental implant planning process, including determining canals, sinuses, fossae, bone length and breadth in areas where teeth are missing. We then compared the information collected by both human evaluators and AI tools. Furthermore, to evaluate the effectiveness of the AI model, the expert conducted the same procedures independently on the testing data, following the identical steps mentioned above without accessing the AI model's results, ensuring an unbiased comparison.

## The proposed 3D deep learning network

**Figure 6:**
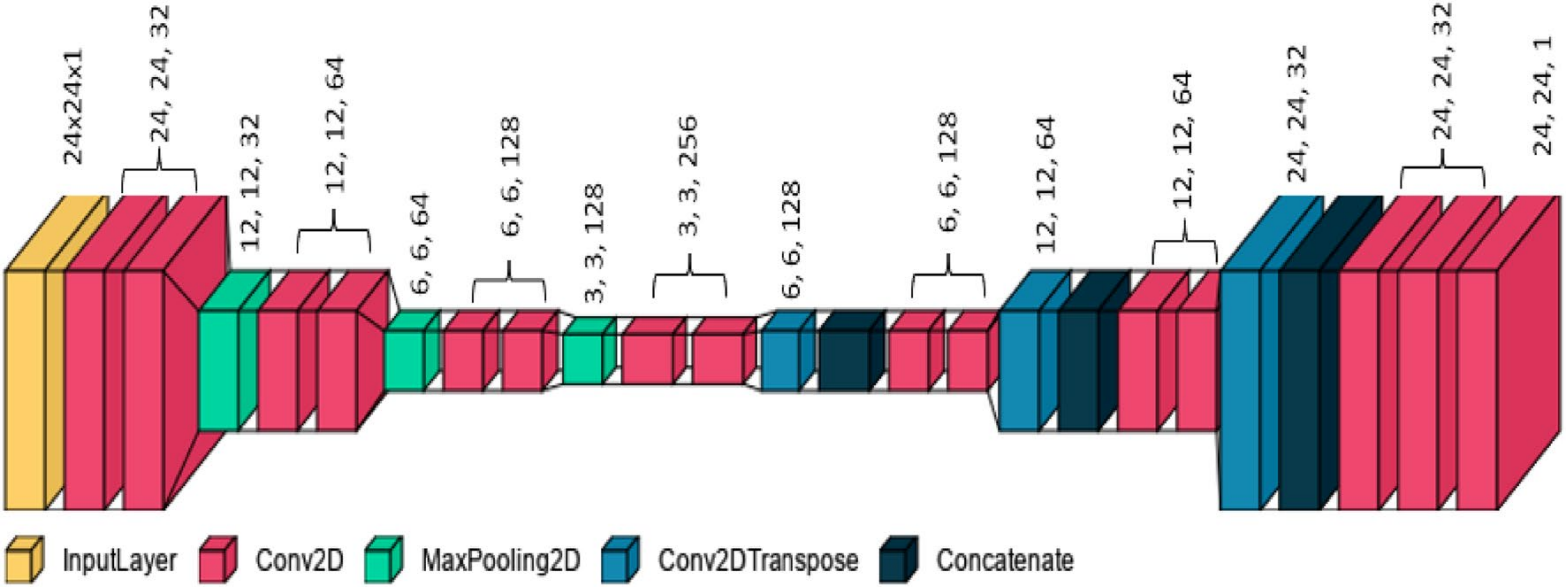
U-net deep learning structure.

To address the problem of missing teeth segmentation in CBCT volumetric data, this study utilized the popular UNET model trained through supervised learning. The structure of our model is depicted in Fig. 6.

U-Net is a popular convolutional neural network (CNN) architecture that has been used for biomedical image segmentation. It captures both local and global contexts by using skip connections. The model requires input images of size 24 × 24 × 1, where 1 represents the number of input channels (e.g., 1 for grayscale images, 3 for RGB images). It is worth mentioning that the original input layer of the Unet model uses high-resolution images. However, resizing the input layer to a smaller size can help mitigate the computational demands while still capturing the relevant information for the task at hand.

**Figure 7:**
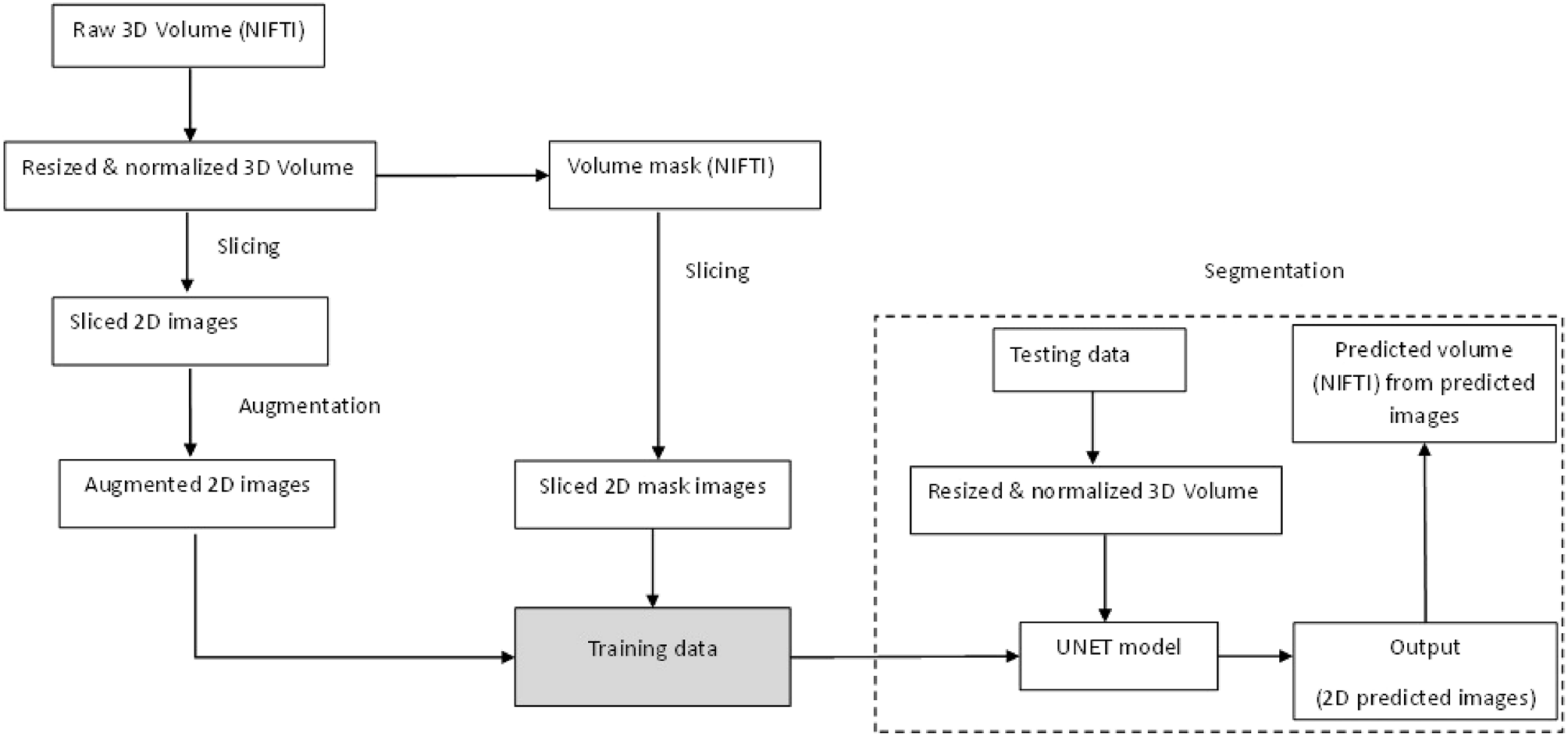
The proposed framework for 3D volumetric data segmentation.

The model starts with a series of convolutional blocks. The number of convolutional blocks in each level of the U-Net model was set to two, and the number of initial features in the first convolutional block was set to 32. Each convolutional block consists of multiple 2D convolutional layers with specified kernel size, activation function, and padding. The number of features in each block increases as the initial features were multiplied by two raised to the power of the current level. Skip connections were created during the downstream path by storing intermediate feature maps in a “skips” dictionary. The current feature map was stored in “skips” before downsampling the feature maps using max pooling. The upstream path starts by using transpose convolutional layers (also known as deconvolutional layers) to upsample the feature maps. The transpose convolutional layers have the same kernel size, activation function, and padding as the convolutional blocks in the downstream path. The feature maps from the corresponding level in the downstream path were concatenated with the upsampled feature maps using the stored skip connections. This process was repeated for each level of four levels in reverse order. The final output layer consists of a 2D convolutional layer with a kernel size of 1 and a sigmoid activation function. The output has the exact spatial dimensions as the input, with each pixel representing the predicted class probabilities. Figure 7 summarizes the proposed framework for 3D volumetric data segmentation. It is essential to highlight that we employed two UNet models in this study to address the distinct tasks of segmentation and prediction more effectively, leveraging the strengths of each model for optimal performance in their respective tasks. By utilizing two separate models, we exploit each model’s specific capabilities and architectures to optimize the performance of the required tasks. These models can be trained independently, allowing for dedicated fine-tuning and optimization for each task.

Furthermore, applying two models can provide more flexibility and modularity in the overall system. It also allows for potential improvements or updates to be made on one model without affecting the other. The raw CBCT volumes are all in the neuroimaging informatics technology initiative (NIfTI or nii) format and are resized and normalized using Python Nibabel library 31. After that, another script takes every volume and mask as input and slices them along the x, y, and z axes. All produced 2D slices/images have the same size and format as the proposed UNET model for training. The augmentation step was done using the ImageDataGenerator class in the Keras API. Once the model is trained and saved, it can predict new unseen volumes. Since the expected input/output of the model are 2D grayscale images, we first sliced the volume to be predicted and fed the slices to the model for prediction. In each iteration, one image is fed to the model, and its predicted 2D mask is saved in a list. This iterates over all predicted 2D images and stacks them together to form the final predicted volume in NIfTI format using the Nibabel library31. We also saved the predicted volume in STL format using the numpy-stl library for visualization.

It is important to highlight that the proposed approach specifically designed to tackle the complex issues associated with implant site segmentation. This method was developed after carefully considering the specific needs and characteristics of dental imaging, which often differ significantly from more general medical image segmentation tasks. The proposed approach has shown high accuracy in dental segmentation tasks, outperforming many existing methods. We have compared it with other techniques in jaw and teeth segmentation and found that it performs well, with high precision and recall rates. This solution is not only effective but also accessible for practitioners in the field, enabling more accurate diagnostics and treatment planning in dental care.

### Ethical considerations

The process starts with obtaining ethical approval to collect CBCT scans. The study protocol received approval from the research ethics committee at Taibah University’s College of Dentistry in Madinah, Saudi Arabia (# TUCDREC/14032021, approved on March 21, 2021). Due to the non-interventional retrospective design and anonymous data assessment, the Research Ethics Committee of Taibah University’s College of Dentistry waived the need for individual informed consent. All methods in this study were conducted following relevant guidelines and regulations.

## Conclusion

In this paper, we proposed a framework for segmenting 3D volumetric data for dental implant planning. We collected CBCT scans from patients who visited Taibah University Dental Hospital (TUDH) between 2018 and 2023 and underwent CBCT imaging. A total of 150 CBCT images were used in this study. The raw CBCT volumes were resized and normalized. We then sliced the volume and its mask with the same size and format. We trained a U-Net deep learning model using these slices, which allowed us to predict new unseen volumes. Since the U-Net model takes 2D images as input/output, we also sliced each volume and fed the slices to the U-Net model to predict the final volume in 3D NIfTI and STL formats. The experimental results demonstrate the excellent performance of the proposed deep learning models in segmenting the region of interest (ROI) and predicting missing teeth. The obtained dice, Jaccard, precision, and recall values were 0.93, 0.88, 0.94, and 0.93, respectively. The proposed models also have the advantage of low computational cost, making it suitable for various therapeutic procedures. However, this study has a few limitations, including the moderate accuracy of the deep learning models for missing tooth bone segmentation, dependence on segmentation software like 3D Slicer, and manual cropping of volumes to isolate the region of interest for missing tooth segmentation. This limitation highlights the need for further research and development in deep learning models for implant planning, as well as advancements in segmentation techniques. Addressing these limitations would help improve the accuracy and efficiency of the implant planning process. Despite this limitation, we hope that this preliminary study will motivate more in-depth research in the future. Increasing the quantity of CBCT images and using appropriate data augmentation tools and network architectural configurations can enhance the quality of the training. Additionally, further research can be conducted on the entire dental implant planning sequence.

### Supplementary Information

Supplementary Information.

## Data Availability

Database CBCT scans were employed as a data source for this investigation. They were gathered in Madinah, Saudi Arabia, at the College of Dentistry’s Educational Hospital between 2018 and 2023. Metadata containing protected health information was scrubbed following the Institutional Review Board’s approval (IRB No. 14032021). Software MeshLab v.2022.02 was used to produce all the images in this article, while Microsoft Office 365 was used to produce the graphics in Figs. 1, 6, and

7. Upon a reasonable request, the corresponding author will provide access to the data supporting the conclusions of this study. The proposed framework for 3D volumetric data segmentation.
