# Supplementary material for: Deep learning-based approach for 3D bone segmentation and prediction of missing tooth region for dental implant planning
Source: Sci Rep. 2024 Jun 16;14:13888. doi: 10.1038/s41598-024-64609-0 (PMC11180661; doi:10.1038/s41598-024-64609-0)
Supplement: Supplementary file 1 — Supplementary Information. [file 41598_2024_64609_MOESM1_ESM.docx]

**Deep Learning-based Approach for 3D Bone Segmentation and Prediction of Missing Tooth Region for Dental Implant Planning**

Mohammed Al-Asali, Ahmed Yaseen Alqutaibi, Mohammed Al-Sarem, and Faisal Saeed

**Supplementary Material**

Our deep learning models were trained on a desktop PC with the following specifications:

1. Intel Core i9 11980K CPU @3.30GHz
2. 32GB DDR4 memory
3. NVidia GeForce RTX 3070 Ti graphics card
4. CUDA 11.8.522
5. CUDNN 11.2.0
6. Python 3.9.0
7. TensorFlow 2.1.0
8. Keras 2.3.1
9. Windows 11: 64-bit

The architecture of our deep learning models is described in detail in Figure A1. The model was trained using a validation split of 0.2. The training session consisted of 200 epochs and took a total of 58 minutes to complete. After the model was trained, it was evaluated using the same settings. During the evaluation, a prediction task using the trained model only required 10 seconds to finish. This suggests that the trained model is capable of quickly generating outputs or making predictions once it has been trained.

__________________________________________________________________________

Layer (type) Output Shape Param # Connected to

=================================================================

input_1 (InputLayer) [(None, 24, 24, 1)] 0 []

conv2d (Conv2D) (None, 24, 24, 32) 320 ['input_1[0][0]']

conv2d_1 (Conv2D) (None, 24, 24, 32) 9248 ['conv2d[0][0]']

max_pooling2d (MaxPooling2D) (None, 12, 12, 32) 0 ['conv2d_1[0][0]']

conv2d_2 (Conv2D) (None, 12, 12, 64) 18496 ['max_pooling2d[0][0]']

conv2d_3 (Conv2D) (None, 12, 12, 64) 36928 ['conv2d_2[0][0]']

max_pooling2d_1 (MaxPooling2D) (None, 6, 6, 64) 0 ['conv2d_3[0][0]']

conv2d_4 (Conv2D) (None, 6, 6, 128) 73856 ['max_pooling2d_1[0][0]']

conv2d_5 (Conv2D) (None, 6, 6, 128) 147584 ['conv2d_4[0][0]']

max_pooling2d_2 (MaxPooling2D) (None, 3, 3, 128) 0 ['conv2d_5[0][0]']

conv2d_6 (Conv2D) (None, 3, 3, 256) 295168 ['max_pooling2d_2[0][0]']

conv2d_7 (Conv2D) (None, 3, 3, 256) 590080 ['conv2d_6[0][0]']

conv2d_transpose (Conv2DTransp (None, 6, 6, 128) 295040 ['conv2d_7[0][0]']

concatenate (Concatenate) (None, 6, 6, 256) 0 ['conv2d_transpose[0][0]', 'conv2d_5[0][0]']

conv2d_8 (Conv2D) (None, 6, 6, 128) 295040 ['concatenate[0][0]']

conv2d_9 (Conv2D) (None, 6, 6, 128) 147584 ['conv2d_8[0][0]']

conv2d_transpose_1 (Conv2DTran (None, 12, 12, 64) 73792 ['conv2d_9[0][0]']

concatenate_1 (Concatenate) (None, 12, 12, 128) 0 ['conv2d_transpose_1[0][0]', 'conv2d_3[0][0]']

conv2d_10 (Conv2D) (None, 12, 12, 64) 73792 ['concatenate_1[0][0]']

conv2d_11 (Conv2D) (None, 12, 12, 64) 36928 ['conv2d_10[0][0]']

conv2d_transpose_2 (Conv2DTran (None, 24, 24, 32) 18464 ['conv2d_11[0][0]']

concatenate_2 (Concatenate) (None, 24, 24, 64) 0 ['conv2d_transpose_2[0][0]', 'conv2d_1[0][0]']

conv2d_12 (Conv2D) (None, 24, 24, 32) 18464 ['concatenate_2[0][0]']

conv2d_13 (Conv2D) (None, 24, 24, 32) 9248 ['conv2d_12[0][0]']

conv2d_14 (Conv2D) (None, 24, 24, 1) 33 ['conv2d_13[0][0]']

=================================================================

Total params: 2,140,065

Trainable params: 2,140,065

Non-trainable params: 0

*Figure A1. Detailed architecture of the proposed deep learning models. (Created by the Keras Python library)*

We selected an additional two CBCT volumes (called volume 2 and volume 3) to further test the proposed models and to ensure the consistency of our reported results. Table A1 shows the performance of our U-NET model 1 for volume 2 and volume 3. Figure A2 and Figure A3 visualize the predicted volumes for volume 2 and volume 3, respectively. Although most of the metrics show a good performance of the model, volume error rates of 15.73% and 16.30% suggest that there is an average deviation in the measured volumes compared to the true or reference volumes. This could be due to various factors such as inherent limitations of the imaging technology, image reconstruction algorithms, image artifacts, or variations in the scanning process.

*Table A1: U-NET model 1 performance results for a sample volumes (volume 2 and volume 3)*

|  | dice | jaccard | precision | recall | fpr | fnr | vs | hd | msd | mdsd | stdsd | hd95 | Volume error rate |
| --- | --- | --- | --- | --- | --- | --- | --- | --- | --- | --- | --- | --- | --- |
| Volume 2 | 0.90 | 0.82 | 0.84 | 0.97 | 0.01 | 0.02 | -0.14 | 3.07 | 0.27 | 0.29 | 0.34 | 0.89 | 15.73% |
| Volume 3 | 0.88 | 0.80 | 0.89 | 0.94 | 0.04 | 0.05 | -0.15 | 3.8 | 0.24 | 0.30 | 0.38 | 0.94 | 16.30% |

| 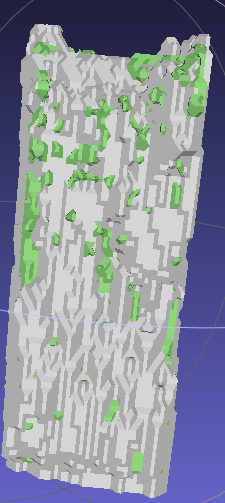 | 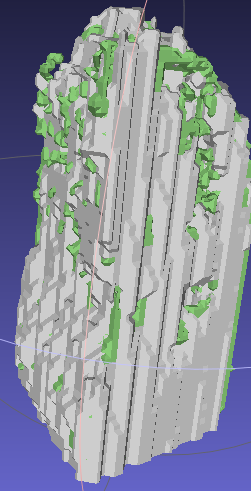 | 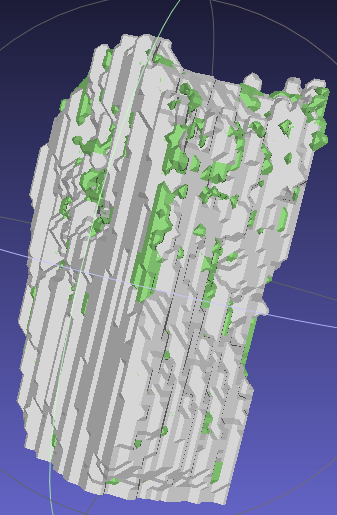 |
| --- | --- | --- |
| a | b | c |
| 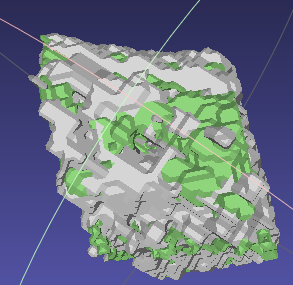 | 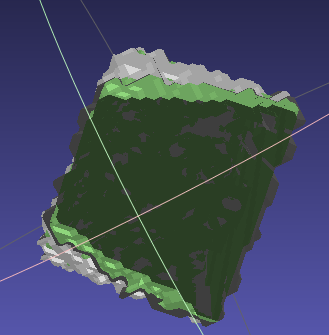 | 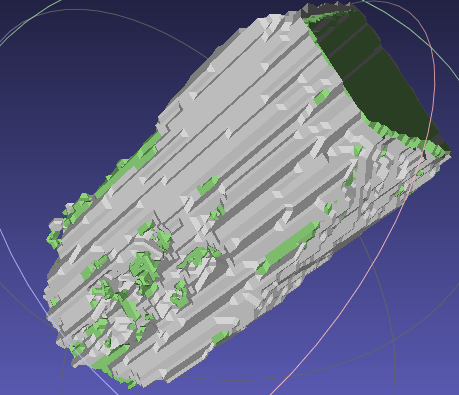 |
| d | e | f |

Figure A2. Side View of the Volume obtained by the first trained model for volume 2: (a, b, c)- Front side view of the volume of tooth bone, d: upper view of the volume, e: bottom view of the volume, f: side view from the bottom of the volume.

| 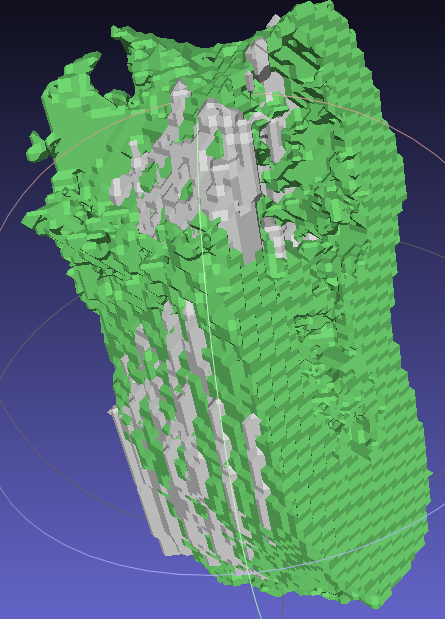 | 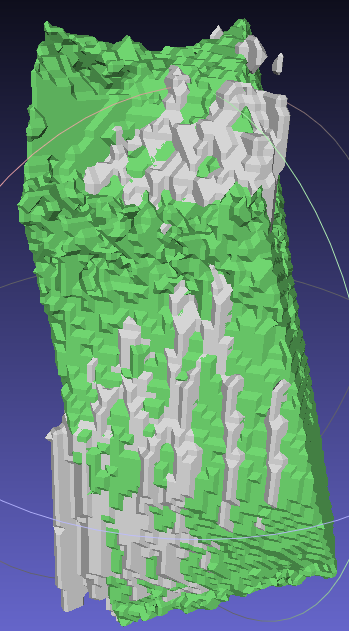 | 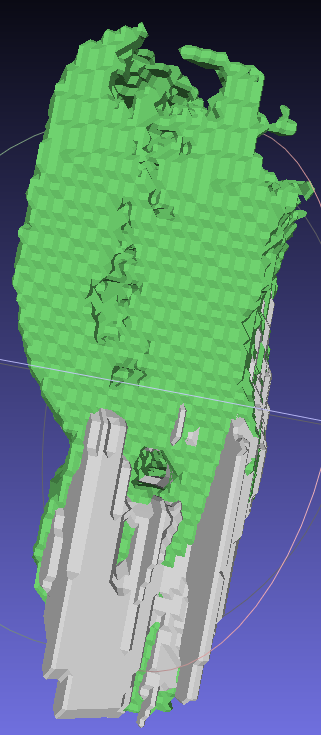 |
| --- | --- | --- |
| a | b | c |
| 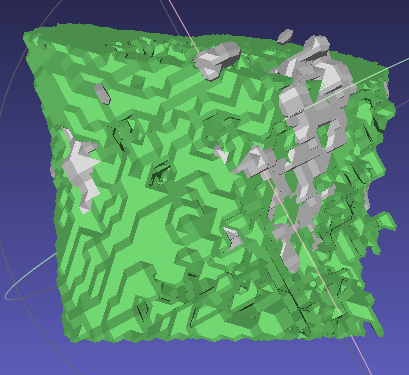 | 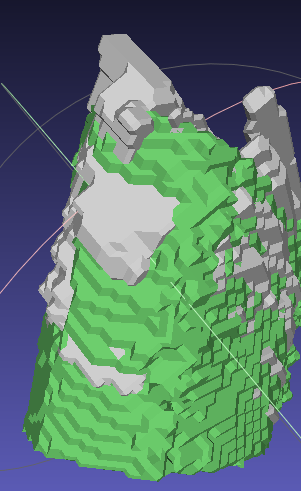 | 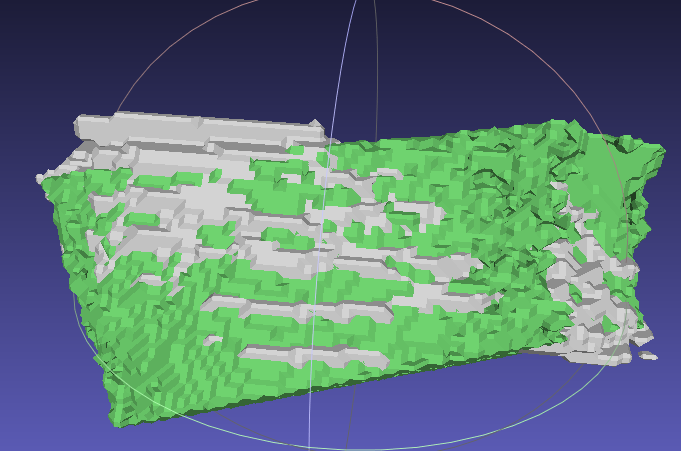 |
| d | e | f |

Figure A3. Side View of the Volume obtained by the first trained model for volume 3: (a, b, c)- Front side view of the volume of tooth bone, d: upper view of the volume, e: bottom view of the volume, f: side view from the bottom of the volume.

Additionally, we tested our proposed U-NET model 2 using volume 2 and volume 3, and the findings are documented in Table A2. The results reveal that the model exhibited superior performance across most of the evaluated metrics. This superiority is further supported by Figure A4 and Figure A5, which display how the predicted volumes matches the ground truth volumes with a low volume error rate.

*Table A2: U-NET model 2 performance results for volume 2 and volume 3*

|  | dice | jaccard | precision | recall | fpr | fnr | vs | hd | msd | mdsd | stdsd | hd95 | Volume error rate |
| --- | --- | --- | --- | --- | --- | --- | --- | --- | --- | --- | --- | --- | --- |
| Volume 2 | 0.89 | 0.81 | 0.94 | 0.85 | 0.00 | 0.14 | 0.09 | 1.92 | 0.16 | 0.00 | 0.24 | 0.60 | 9.04% |
| Volume 3 | 0.94 | 0.88 | 0.93 | 0.94 | 0.00 | 0.05 | -0.01 | 2.47 | 0.17 | 0.00 | 0.29 | 0.67 | 4.66% |

| 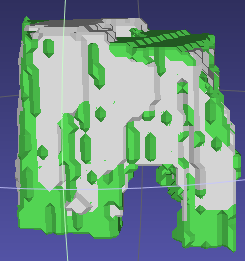 | 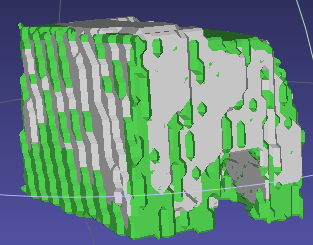 | 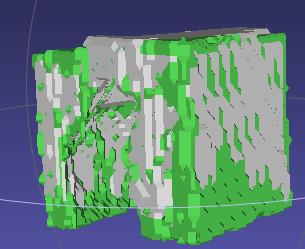 |
| --- | --- | --- |
| a | b | c |
| 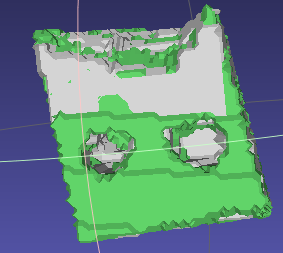 | 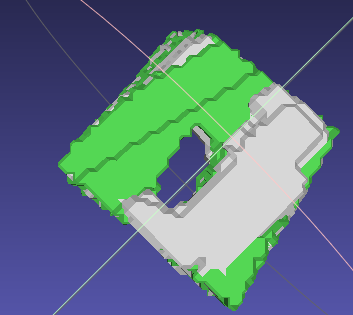 | 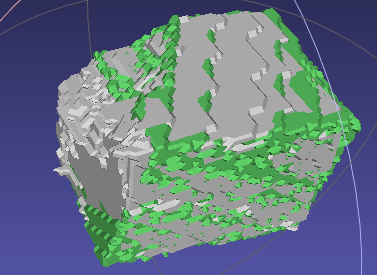 |
| d | e | f |

Figure A4. Side View of the Volume obtained by the second trained model for volume 2: (a, b, c)- Front side view of the volume of tooth bone, d: upper view of the volume, e: bottom view of the volume, f: side view from the bottom of the volume.

| 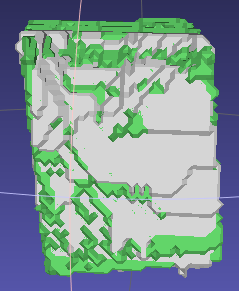 | 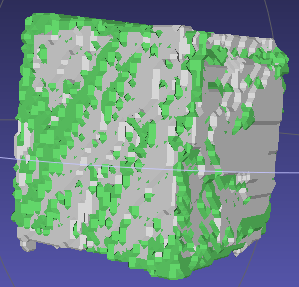 | 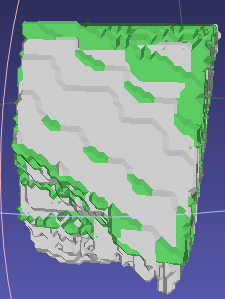 |
| --- | --- | --- |
| a | b | c |
| 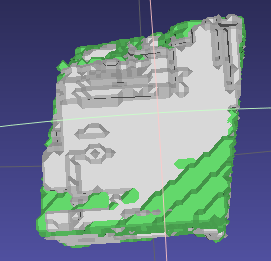 | 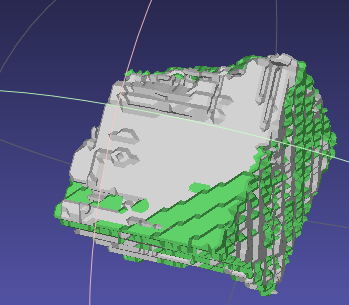 | 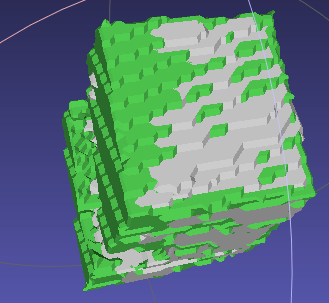 |
| d | e | f |

Figure A5. Side View of the Volume obtained by the second trained model for volume 3: (a, b, c)- Front side view of the volume of tooth bone, d: upper view of the volume, e: bottom view of the volume, f: side view from the bottom of the volume.
